# Supplementary material for: Lipid Digestibility and Polyphenols Bioaccessibility of Oil-in-Water Emulsions Containing Avocado Peel and Seed Extracts as Affected by the Presence of Low Methoxyl Pectin
Source: Foods. 2021 Sep 16;10(9):2193. doi: 10.3390/foods10092193 (PMC8466070; doi:10.3390/foods10092193)
Supplement: Supplementary file 1 [file foods-10-02193-s001.zip › foods-1336869-supplementary.pdf]

## SUPPLEMENTARY TABLES

**S1.** Concentration of the phenolic compounds from avocado peel (AP) released after the simulated intestinal digestion of oil-in-water emulsions, with or without low methoxyl pectin (LMP).

| Phenolic compounds | Digested emulsion | | | | Released fraction | | | |
|---|---|---|---|---|---|---|---|---|
| | Containing LMP | | Without LMP | | Containing LMP | | Without LMP | |
| | Blank emulsion | Emulsion with AP extract | Blank emulsion | Emulsion with AP extract | Blank emulsion | Emulsion with AP extract | Blank emulsion | Emulsion with AP extract |
| Protocatechuic acid glucoside | n.d. | n.d. | n.d. | 7.67 | n.d. | n.d. | n.d. | n.d. |
| Hydroxytyrosol | n.d. | n.d. | n.d. | n.d. | n.d. | 0.39 ± 0.26 | n.d. | 0.64 |
| Hydroxytyrosol glucoside | n.d. | n.d. | n.d. | n.d. | n.d. | n.d. | n.d. | n.d. |
| Hydroxytyrosol glucoside arabinoside | n.d. | n.d. | n.d. | n.d. | n.d. | n.d. | n.d. | n.d. |
| Tyrosol glucoside | n.d. | n.d. | n.d. | n.d. | n.d. | n.d. | n.d. | n.d. |
| Tyrosol glucoside arabinoside | n.d. | n.d. | n.d. | n.d. | n.d. | n.d. | n.d. | n.d. |
| Salidroside | n.d. | n.d. | n.d. | n.d. | n.d. | n.d. | n.d. | n.d. |
| P-coumaric acid | 0.50 ± 0.00 | 17.1 ± 0.26 | n.d. | 60.1 | 0.81 ± 0.54 | 46.4 ± 5.77 | 4.79 | 71.7 |
| Coumaroylquinic acid | n.d. | 0.42 ± 0.39 | n.d. | 3.77 | n.d. | 1.77 ± 0.12 | n.d. | 4.30 |
| Caffeic acid | 0.63 ± 0.50 | 15.3 ± 0.79 | n.d. | 62.8 | 0.73 ± 0.23 | 36.4 ± 0.68 | 9.65 | 73.1 |
| Caffeoylshikimic acid | n.d. | 0.17 ± 0.03 | n.d. | 1.10 | n.d. | 0.53 ± 0.26 | n.d. | 10.5 |
| Caffeic acid glucoside | n.d. | 0.38 ± 0.21 | n.d. | 1.92 | n.d. | 0.33 ± 0.24 | n.d. | 0.86 |
| 3-*O*-caffeoylquinic acid | 3.39 ± 1.52 | 4.96 ± 2.28 | n.d. | 21.8 | n.d. | 327.0 ± 28.7 | n.d. | 0.84 |
| 4-*O*-caffeoylquinic acid | n.d. | 2.25 ± 0.34 | n.d. | 8.43 | n.d. | 6.76 ± 1.34 | n.d. | 13.0 |
| 5-*O*-caffeoylquinic acid | 2.56 ± 2.47 | 45.0 ± 7.36 | n.d. | 232.6 | n.d. | 101.2 ± 7.36 | n.d. | 281.5 |
| (Iso) ferulic acid | n.d. | 3.05 ± 0.50 | n.d. | 10.2 | 0.41 ± 0.00 | 7.96 ± 0.47 | n.d. | 10.2 |
| Dihydroferulic acid | n.d. | 0.32 ± 0.09 | n.d. | 0.60 | n.d. | 0.81 ± 0.01 | n.d. | 1.29 |
| Ferulic acid glucoside | n.d. | 2.23 ± 0.47 | n.d. | 11.4 | n.d. | 7.19 ± 1.58 | n.d. | 17.8 |
| Dihydroferulic acid glucoside | n.d. | 0.49 ± 0.15 | n.d. | 1.06 | n.d. | 0.85 ± 0.28 | n.d. | 2.59 |
| 4-*O*-feruoylquinic acid | n.d. | 1.14 ± 0.34 | n.d. | 2.83 | n.d. | 2.88 ± 0.02 | n.d. | 4.69 |
| 5-*O*-feruoylquinic acid | n.d. | n.d. | n.d. | n.d. | n.d. | 3.29 ± 0.19 | n.d. | 6.25 |
| 6-*O*-feruoylquinic acid | n.d. | 2.19 ± 0.12 | n.d. | 6.58 | n.d. | 5.47 ± 0.13 | n.d. | 8.60 |
| ***Total phenolic acids*** | *7.5 ± 4.46* | *95.01 ± 11.70* | *n.d.* | *425.19* | *5.67 ± 0.87* | *124.31 ± 12.12* | *14.44* | *507.86* |
| Catechin | 2.92 ± 2.88 | 71.4 ± 3.46 | n.d. | 518.3 | n.d. | 63.2 ± 6.63 | n.d. | 175.8 |
| Epicatechin | 6.60 ± 7.40 | 810.1 ± 100.9 | n.d. | 271.8 | n.d. | 815.6 ± 88.5 | n.d. | 2038.4 |
| Epigallocatechin | n.d. | n.d. | n.d. | 10.8 | n.d. | n.d. | n.d. | n.d. |
| Catechin glucoside | n.d. | n.d. | n.d. | 11.5 | n.d. | n.d. | n.d. | n.d. |
| Epicatechin glucoside | n.d. | 32.8 ± 9.58 | n.d. | 177.9 | n.d. | 39.0 ± 9.20 | n.d. | 115.2 |
| Dimer (type A) | n.d. | 72.4 ± 16.6 | n.d. | 368.7 | n.d. | 42.0 ± 5.58 | n.d. | 122.2 |
| Dimer (type B) | n.d. | 774.6 ± 57.9 | n.d. | 4616.0 | n.d. | 813.9 ± 34.7 | n.d. | 2218.2 |
| Trimer (type A) | 1.93 ± 0.66 | 49.0 ± 15.5 | n.d. | 481.7 | n.d. | 45.8 ± 5.13 | n.d. | 143.3 |

| | | | | | | | | |
|---|---|---|---|---|---|---|---|---|
| Trimer (type B) | n.d. | 173.9 ± 22.4 | n.d. | 2098.7 | n.d. | 208.1 ± 3.55 | n.d. | 277.7 |
| Tetramer | n.d. | 4.53 ± 4.41 | n.d. | 118.1 | n.d. | 13.6 ± 0.12 | n.d. | 61.8 |
| Pentamer | n.d. | n.d. | n.d. | n.d. | n.d. | n.d. | n.d. | n.d. |
| Hexamer | n.d. | n.d. | n.d. | 38.4 | n.d. | n.d. | n.d. | n.d. |
| Quercetin | n.d. | 2.50 ± 1.13 | n.d. | 4.15 | n.d. | n.d. | n.d. | 3.78 |
| Quercetin arabinoside | n.d. | 0.70 ± 0.06 | n.d. | 2.72 | n.d. | n.d. | n.d. | 3.33 |
| Quercetin rhamnoside | n.d. | 0.83 ± 0.54 | n.d. | 2.06 | n.d. | n.d. | n.d. | 2.06 |
| Quercetin glucoside | n.d. | 34.0 ± 10.4 | n.d. | 131.7 | n.d. | 22.2 ± 5.61 | n.d. | 49.8 |
| Quercetin glucuronide | n.d. | 13.6 ± 0.47 | n.d. | 76.4 | n.d. | 27.5 ± 1.26 | n.d. | 74.3 |
| Quercetin glucoside arabinoside | n.d. | 257.8 ± 96.1 | n.d. | 913.7 | n.d. | 244.8 ± 45.8 | n.d. | 568.1 |
| Quercetin rutinoside | n.d. | 11.2 ± 1.36 | n.d. | 34.6 | n.d. | 17.9 ± 6.01 | n.d. | 29.9 |
| Quercetin diglucoside | n.d. | 164.4 ± 82.8 | n.d. | 396.3 | n.d. | 164.6 ± 22.4 | n.d. | 855.5 |
| Multinoside A | n.d. | 43.2 ± 2.28 | n.d. | 103.8 | n.d. | 37.9 ± 2.53 | n.d. | 132.3 |
| Kaempferol glucoside | n.d. | 7.62 ± 2.43 | n.d. | 23.3 | n.d. | n.d. | n.d. | n.d. |
| Kampeferol glucosido arabinoside | n.d. | 84.4 ± 7.22 | n.d. | 148.4 | n.d. | 60.4 ± 14.1 | n.d. | 183.4 |
| Kampeferol rutinoside | n.d. | 29.0 ± 1.95 | n.d. | 59.1 | n.d. | 25.6 ± 2.78 | n.d. | 81.7 |
| Kampeferol diglucoside | n.d. | 103.5 ± 41.8 | n.d. | 159.1 | n.d. | 174.8 ± 2.83 | n.d. | 317.9 |
| Isorhamnetin arabinoside | n.d. | 0.16 ± 0.00 | n.d. | 0.33 | n.d. | n.d. | n.d. | n.d. |
| Isorhamnetin glucuronide | n.d. | 2.58 ± 0.37 | n.d. | 13.2 | n.d. | 6.96 ± 3.33 | n.d. | 25.0 |
| Isorhamnetin arabinoside glucoside | n.d. | 0.61 ± 0.20 | n.d. | 1.51 | n.d. | 0.52 ± 0.01 | n.d. | 1.65 |
| Isorhamnetin derivative | n.d. | 1.29 ± 0.88 | n.d. | 4.01 | n.d. | 2.39 ± 0.59 | n.d. | 5.48 |
| ***Total Flavonoids*** | *11.4 ± 10.9* | *2745.93 ± 291.56* | *n.d.* | *13997.3* | *n.d.* | *2827.06 ± 193.95* | *n.d.* | *7487.1* |
| Penstemide | n.d. | 9.08 ± 0.61 | n.d. | 58.2 | n.d. | 22.3 ± 5.70 | n.d. | 40.9 |
| Cinchonain | n.d. | 4.53 ± 3.02 | n.d. | 28.8 | n.d. | n.d. | n.d. | n.d. |
| nudiposide | n.d. | 1.64 ± 0.29 | n.d. | 3.84 | n.d. | 4.11 ± 0.82 | n.d. | 7.23 |
| ***Total terpenes*** | *n.d.* | *15.2 ± 3.36* | *n.d.* | *90.8* | *n.d.* | *26.4 ± 4.88* | *n.d.* | *48.1* |
| ***TOTAL (mg/100 g)*** | *24.5 ± 17.1* | *2856.1 ± 185.6* | *6.89* | *14520.0* | *2.93 ± 1.30* | *3402.7 ± 158.3* | *27.0* | *8836.3* |
| ***TOTAL (g/100 g)*** | *0.02 ± 0.02* | *2.86 ± 0.19* | *0.01* | *14.5* | *0.00 ± 0.00* | *3.40 ± 0.16* | *0.03* | *8.84* |

Data expressed as mg individual phenolic compound per 100 g of sample ± standard deviation.

**S2.** Concentration of the phenolic compounds from avocado seed (AS) released after the simulated intestinal digestion of oil-in-water emulsions, with or without low methoxyl pectin (LMP).

| Phenolic compounds | Digested emulsion | | | | Released fraction | | | |
|---|---|---|---|---|---|---|---|---|
| | Containing LMP | | Without LMP | | Containing LMP | | Without LMP | |
| | Blank emulsion | Emulsion with AS extract | Blank emulsion | Emulsion with AS extract | Blank emulsion | Emulsion with AS extract | Blank emulsion | Emulsion with AS extract |
| *p*-hydroxybenzoic acid | 0.76 ± 0.33 | 1.16 ± 0.11 | 0.69 | 2.90 | 0.98 ± 0.36 | 1.89 ± 0.14 | 1.26 | 3.37 |
| Protocatechuic acid | n.d. | 3.88 ± 0.03 | n.d. | 12.0 | n.d. | n.d. | n.d. | n.d. |
| Protocatechuic acid glucoside | n.d. | n.d. | n.d. | n.d. | n.d. | n.d. | n.d. | n.d. |
| Hydroxytyrosol | n.d. | 0.63 ± 0.36 | n.d. | 2.56 | n.d. | 1.09 ± 0.63 | n.d. | 3.75 |
| Hydroxytyrosol glucoside | n.d. | 2.34 ± 0.06 | n.d. | 12.2 | n.d. | 4.14 ± 0.15 | n.d. | 11.0 |
| Hydroxytyrosol glucoside arabinoside | n.d. | 8.79 ± 1.55 | n.d. | 37.9 | n.d. | 10.8 ± 1.90 | n.d. | 43.2 |
| Tyrosol glucoside | n.d. | n.d. | n.d. | n.d. | n.d. | n.d. | n.d. | n.d. |
| Tyrosol glucoside arabinoside | n.d. | 23.0 ± 3.20 | n.d. | 119.3 | n.d. | n.d. | n.d. | n.d. |
| Salidroside | 5.24 ± 1.33 | 116.9 ± 4.48 | n.d. | 1013.1 | n.d. | 78.0 ± 0.43 | n.d. | 194.1 |
| Hydroxysalidrosid | n.d. | 9.40 ± 5.89 | n.d. | 67.7 | n.d. | 2.34 ± 3.31 | n.d. | n.d. |
| P-coumaric acid | 0.49 ± 0.00 | 2.95 ± 0.08 | n.d. | 7.72 | 081 ± 0.54 | 6.00 ± 0.54 | 0.48 | 9.12 |
| Coumaroylquinic acid | n.d. | 4.47 ± 0.20 | n.d. | 23.4 | n.d. | 14.6 ± 0.14 | n.d. | 30.3 |
| Caffeic acid | 0.63 ± 0.46 | 15.2 ± 0.03 | n.d. | 52.2 | 0.73 ± 0.23 | 23.5 ± 2.39 | 0.96 | 55.0 |
| Dihydrocaffeic acid | n.d. | n.d. | n.d. | n.d. | n.d. | n.d. | n.d. | n.d. |
| Caffeoylshikimic acid | n.d. | n.d. | n.d. | n.d. | n.d. | n.d. | n.d. | n.d. |
| Caffeic acid glucoside | n.d. | n.d. | n.d. | 1.53 | n.d. | 0.72 ± 0.02 | n.d. | 1.67 |
| Dihydrocaffeic acid glucoside | n.d. | n.d. | n.d. | 2.20 | n.d. | 0.96 ± 0.12 | n.d. | 1.6 |
| 3-*O*-caffeoylquinic acid | 3.39 ± 1.52 | 95.3 ± 7.19 | n.d. | 669.3 | n.d. | 199.9 ± 54.7 | n.d. | 748.1 |
| 5-*O*-caffeoylquinic acid | n.d. | 85.6 ± 10.3 | n.d. | 310 | n.d. | 185 ± 12.7 | n.d. | 630 |
| 4-*O*-caffeoylquinic acid | 2.56 ± 2.47 | 69.5 ± 2.45 | n.d. | 287.9 | n.d. | 146.3 ± 20.7 | n.d. | 332.5 |
| (Iso) ferulic acid | n.d. | n.d. | n.d. | n.d. | 0.41 ± 0.00 | 0.58 ± 0.09 | n.d. | 0.64 |
| Ferulic acid glucoside | n.d. | 1.20 ± 0.33 | n.d. | 6.34 | n.d. | 2.37 ± 1.60 | n.d. | 5.30 |
| Dihydroferulic acid glucoside | n.d. | n.d. | n.d. | n.d. | n.d. | n.d. | n.d. | n.d. |
| 3-*O*-feruoylquinic acid | n.d. | 0.86 ± 0.15 | n.d. | 3.49 | n.d. | 2.46 ± 0.06 | n.d. | 4.37 |
| 5-*O*-feruoylquinic acid | n.d. | n.d. | n.d. | n.d. | n.d. | 1.59 ± 0.01 | n.d. | 2.85 |
| 4-*O*-feruoylquinic acid | n.d. | 2.62 ± 0.02 | n.d. | 12.5 | n.d. | 5.64 ± 0.18 | n.d. | 14.6 |
| ***Total phenolic acids*** | *10.16 ± 6.11* | *366.4 ± 27.60* | *0.69* | *2365,4* | *2.52 ± 1.13* | *521.5 ± 88.38* | *2.7.* | *1530.90* |
| Catechin | 2.92 ± 2.88 | 55.0 ± 5.06 | n.d. | 574.8 | n.d. | 54.4 ± 8.71 | n.d. | 233.6 |
| Epicatechin | 6.60 ± 7.40 | 201.6 ± 49.1 | n.d. | 1030.6 | n.d. | 122.6 ± 10.5 | n.d. | 433.7 |
| Epigallocatechin | n.d. | n.d. | n.d. | n.d. | n.d. | n.d. | n.d. | n.d. |
| Epicatequina galato | n.d. | 1.09 ± 0.63 | n.d. | 7.22 | n.d. | 7.56 ± 0.45 | n.d. | 16.6 |

| | | | | | | | | |
|---|---|---|---|---|---|---|---|---|
| Catechin glucoside | n.d. | 11.0 ± 3.66 | n.d. | 60.5 | n.d. | 16.4 ± 2.19 | n.d. | 56.6 |
| Epicatechin glucoside | n.d. | 20.6 ± 4.56 | n.d. | 106.0 | n.d. | n.d. | n.d. | n.d. |
| Dimer (type A) | n.d. | 26.6 ± 2.42 | n.d. | 129.5 | n.d. | 8.53 ± 2.57 | n.d. | 49.3 |
| Dimer (type B) | n.d. | 88.7 ± 1.30 | n.d. | 754.8 | n.d. | 70.4 ± 11.1 | n.d. | 391.9 |
| Trimer (type A) | 1.93 ± 0.66 | 306.6 ± 5.77 | n.d. | 1446.4 | n.d. | 154.3 ± 24.1 | n.d. | 734.6 |
| Trimer (type B) | n.d. | 7.20 ± 0.37 | n.d. | 65.6 | n.d. | 3.78 ± 0.08 | n.d. | 31.2 |
| Tetramer | n.d. | 0.52 ± 0.73 | n.d. | 6.26 | n.d. | n.d. | n.d. | 7.39 |
| Quercetin glucoside | n.d. | 3.63 ± 1.38 | n.d. | 11.2 | n.d. | 2.19 ± 0.00 | n.d. | 7.56 |
| Quercetin diglucoside | n.d. | 3.40 ± 0.63 | n.d. | 11.1 | n.d. | 4.26 ± 0.23 | n.d. | 8.74 |
| Quercetin glucuronide | n.d. | n.d. | n.d. | n.d. | n.d. | 0.57 ± 0.81 | n.d. | n.d. |
| Quercetin glucoside arabinoside | n.d. | n.d. | n.d. | n.d. | n.d. | 2.30 ± 0.22 | n.d. | 2.57 |
| Quercetin rutinósido | n.d. | n.d. | n.d. | n.d. | n.d. | n.d. | n.d. | n.d. |
| Kaempferol glucoside | n.d. | n.d. | n.d. | n.d. | n.d. | n.d. | n.d. | n.d. |
| Kampeferol glucosido arabinoside | n.d. | n.d. | n.d. | n.d. | n.d. | 1.44 ± 2.03 | n.d. | n.d. |
| *Total kaempferol derivates* | n.d. | n.d. | n.d. | n.d. | n.d. | 1.44 ± 2.03 | n.d. | n.d. |
| Isorhamnetin arabinoside | n.d. | 1.77 ± 0.59 | n.d. | 3.95 | n.d. | 0.96 ± 0.08 | n.d. | 2.93 |
| ***Total flavonoids*** | *11.4. ± 10.9* | *727.8 ± 76.2* | *0.69* | *4207,95* | *n.d.* | *498.17 ± 60.93* | *n.d.* | *1976.73* |
| Penstemide | n.d. | 2.73 ± 0.11 | n.d. | 9.60 | n.d. | 9.08 ± 1.03 | n.d. | 21.8 |
| ***Total terpenes*** | n.d. | 2.73 ± 0.10 | n.d. | 9.60 | n.d. | 9.08 ± 1.03 | n.d. | 21.8 |
| ***TOTAL (mg/kg)*** | *21.56 ± 17.01* | *1097.30 ± 103.90* | *0.69* | *6582.95* | *2.52 ± 1.13* | *1028.75 ± 150.34* | *2.70* | *3529.43* |
| ***TOTAL (g/kg)*** | *0.02 ± 0.02* | *1.10 ± 0.10* | *0.00* | *6.58* | *0.00 ± 0.00* | *1.03 ± 0.15* | *0.00* | *3.53* |

Data expressed as mg individual phenolic compound per 100 g of sample ± standard deviation.
